# Screening of “Cry for Help” Signals from *Angelica sinensis* Induced by *Fusarium solani* and Their Potential for Biological Control

**DOI:** 10.3390/metabo16060385

**Published:** 2026-06-02

**Authors:** Tianpeng Xie, Qi Ding, Linhua Yang, Jingyi Wang, Jingxian Wei, Xiaoxue Du, Ling Jin

**Affiliations:** 1 School of Pharmacy, Gansu University of Chinese Medicine, Lanzhou 730000, China; xtp@gszy.edu.cn (T.X.); kjc@gszy.edu.cn (Q.D.); ylh057@gszy.edu.cn (L.Y.); wangjingyi0402@gszy.edu.cn (J.W.); wjx0612@gszy.edu.cn (J.W.); dxx2026@gszy.edu.cn (X.D.); 2 Longyao Industry Innovation Research Institute, Gansu University of Chinese Medicine, Lanzhou 730000, China

**Keywords:** root rot, *Fusarium solani*, plant “cry for help” theory, metabolomics, microbiomics, taurine, methyl cinnamate

## Abstract

Background: Root rot caused by *Fusarium solani* is a devastating disease in *Angelica sinensis* (danggui), leading to severe yield and quality losses. Sustainable control strategies are urgently needed. According to the plant “cry for help” theory, plants under pathogen attack may recruit beneficial microbes via root exudates. However, whether *A. sinensis* employs this strategy against *F. solani* remains unknown. This study aimed to identify potential “cry for help” metabolites and evaluate their biocontrol potential. Methods: LC-MS analysis revealed that *F. solani* infection significantly altered the metabolic profiles of both *A. sinensis* roots and rhizosphere soil. Results: Comparative analysis identified seven metabolites specifically upregulated in infected plants but not detected in the pathogen, including taurine, oxoadipic acid, quinolinic acid, 6-phosphogluconic acid, methyl cinnamate, 2-phenylethanol, and (R)-3-hydroxybutyric acid. Exogenous application of these seven metabolites revealed that taurine and methyl cinnamate significantly alleviated disease symptoms, improved plant growth (root length, biomass), and enhanced the activities of key defense enzymes (peroxidase, POD, phenylalanine ammonia-lyase, PAL, lipoxygenase, LOX, polyphenol oxidase, PPO). Furthermore, taurine and methyl cinnamate reshaped the rhizosphere microbiome. The incidence of root rot was reduced by 51.3% and 50.8%, respectively. Taurine enriched *actinobacteria* (e.g., *Paeniglutamicibacter*) and reduced the relative abundance of pathogenic *Ascomycota* fungi, while methyl cinnamate markedly enriched the nitrogen-fixing bacterium *Azotobacter* and the saprophytic fungus *Schizothecium*. Crucially, both treatments significantly suppressed the proliferation of *F. solani* in the rhizosphere. Conclusions: Our findings demonstrate for the first time that *A. sinensis* activates a “cry for help” response upon attack by *F. solani*, with taurine and methyl cinnamate preliminarily identified as key signaling metabolites that can directly or indirectly inhibit the development of *A. sinensis* root rot. These compounds enhance plant resistance and recruit beneficial microorganisms, offering a novel and promising ecological strategy for the green control of *A. sinensis* root rot.

## 1. Introduction

*Angelica sinensis* (Oliv.) Diels, a perennial herbaceous plant belonging to the family Apiaceae and the genus Angelica, is primarily cultivated in Min County, Weiyuan, and other regions of Gansu Province, China [1,2]. It thrives in high-altitude cold climates, typically at elevations of 1500–3000 m, requiring low temperatures, long-day conditions, and well-drained, fertile sandy loam soils [3]. As a bulk and commonly used traditional Chinese medicinal herb, it possesses significant medicinal and economic value, playing an indispensable role in clinical applications, healthcare products, and the agricultural economy of local planting areas. Root rot caused by soil-borne pathogenic fungi currently represents a major bottleneck limiting both the yield and quality of *A. sinensis* [4]. Research has identified that root rot in *A. sinensis* from Weiyuan, Gansu, is predominantly caused by *Fusarium solani* [5]. Its infection leads to root decay, vascular necrosis, and even whole plant death, resulting in substantial economic losses. Moreover, *F. solani* not only causes direct tissue damage but also produces mycotoxins, which contribute to disease severity and may affect the quality of medicinal materials. At present, the control of *A. sinensis* root rot remains dominated by chemical fungicides, such as carbendazim, mancozeb, and triazole agents. While these chemicals exhibit rapid efficacy, their long-term overuse has triggered severe drawbacks including pesticide accumulation in medicinal materials, environmental contamination, soil microbial dysbiosis, and rapid emergence of drug-resistant pathogens [6,7]. Consequently, chemical control remains the default practice despite its well-recognized drawbacks, making it urgently necessary to explore environmentally friendly and sustainable green control strategies. In addition, field experiments showed that the combined application of chemical fertilizer (CF) and a novel biological agent (BICF) significantly reduced the incidence of Fusarium root rot of *A. sinensis* from 30.56% (CF group) to 12.4% (BICF group). The disease incidence in the combined treatment of organic fertilizer and the new biological agent was only 11% [8]. These findings confirmed that the novel biological agent exhibits effective control against angelica root rot. However, the underlying mechanism remains unclear, especially whether *A. sinensis* can secrete specific metabolites to recruit beneficial microorganisms under pathogen stress. The present study aimed to address the above issues, so as to provide a theoretical basis for the development and application of microbial agents for the prevention and control of angelica root rot.

It is reported that when plants are infected by pathogens, they can secrete specific signaling metabolites by adjusting their metabolic networks. These substances can directly inhibit pathogen growth, activate the plant’s own defense systems, or recruit beneficial microorganisms to form a disease-suppressive microecology. This theory is known as the plant “cry for help” theory [9]. For instance, elevated levels of malic acid were detected in the root exudates of *Arabidopsis thaliana* infected by *Pseudomonas syringae*, and it acts as a chemoattractant to recruit the biocontrol bacterium *Bacillus subtilis* to colonize the roots [10]. Maize (*Zea mays* L.) secretes benzoxazinoids, which can recruit the plant-growth-promoting bacterium *Pseudomonas putida* in the rhizosphere to resist damage from Chlorobi bacterial infection [11]. However, little is known about whether *A. sinensis* produces “cry for help” metabolites under *F. solani* stress, which limits the development of biocontrol technologies based on the plant’s own defenses. Analyzing the plant’s “cry for help” signals under specific pathogen stress is crucial for understanding plant–microbe interaction mechanisms and for developing disease eco-regulation technologies based on signaling molecules.

Therefore, using *A. sinensis* from Weiyuan, Gansu, as material and building upon previous research, this study conducted the following investigations: (1) characterize the metabolic profile of *F. solani*; (2) compare the metabolomic differences between *F. solani*, healthy *A. sinensis* roots, and diseased *A. sinensis* roots to screen for potential “cry for help” metabolites; and (3) validate the effects of exogenously applying potential “cry for help” metabolites on *A. sinensis* growth phenotype, disease resistance, defense enzyme activities, and their regulatory roles on the community structures of rhizosphere bacteria and fungi. The findings aim to preliminarily reveal the interaction mechanism between *A. sinensis* and *F. solani*, providing a theoretical basis and practical guidance for developing novel biocontrol approaches against *A. sinensis* root rot by utilizing the plant’s own “cry for help” signals.

## 2. Materials and Methods

### 2.1. Isolation and Culture of the Pathogen

*F. solani* was isolated and purified from *A. sinensis* plants exhibiting typical root rot symptoms. Diseased plants were collected from a field in Zhixia Village, Shenjiatan, Huichuan Town, Weiyuan County, Dingxi City, Gansu Province (35°05′ N, 103°97′ E). The local altitude is 2450 m, with an annual average temperature of 5 °C. The coldest month (January) averages −12 °C, while the hottest month (July) averages 15 °C. The annual average precipitation is 566.4 mm [12].

Small tissue pieces (0.5 cm × 0.5 cm) were excised from the junction between diseased and healthy tissue on infected *A. sinensis* roots. The pieces were surface-sterilized by immersion in 75% ethanol for 30 s, followed by immersion in 5% sodium hypochlorite solution for 5 min each, and then rinsed three times with sterile distilled water. Residual moisture was removed using sterile filter paper. The sterilized tissue pieces were transferred onto Potato Dextrose Agar (PDA) medium, with three pieces placed in each Petri dish. Cultures were incubated at 25 °C and observed regularly. After colony emergence, a 0.5 cm × 0.5 cm mycelial plug was excised using an inoculation needle and transferred to fresh PDA medium for purification under the same conditions. The purified strain was identified as *F. solani* based on colony and conidial morphology and ITS sequence analysis, which was confirmed in our previous study. The resulting pure, single colonies were subsequently stored at 4 °C for future use [5].

### 2.2. Establishment of the Diseased Plant Model for A. sinensis Root Rot

Healthy *A. sinensis* seedlings were purchased from an agricultural market in Weiyuan County, Dingxi City, Gansu Province. Prior to planting, two-year-old *A. sinensis* plants of uniform size (root diameter approximately 0.55 cm) and weight (approximately 100 g per 100 seedlings) were selected. The plants were rinsed with sterile water for 30 min, followed by surface sterilization via immersion in 70% ethanol and 5% sodium hypochlorite solution for 5 min. Subsequently, the plants were rinsed several times with sterile water to remove residual disinfectants. The sterilized seedlings were then planted individually in sterilized pots (10 cm in diameter, 15 cm in height) containing sterilized soil, with one plant per pot. The isolated and purified pathogenic fungus was cultured on PDA medium for 7 days. Then, 20 mL of sterile water was added to the culture plate, and mycelia were scraped using a sterile rod. The suspension was filtered through two layers of sterile gauze to prepare a spore suspension. The concentration of the resulting inoculum was adjusted to 3.5 × 10^8^ CFU/mL, which was determined as the optimal concentration for establishing the disease model in preliminary studies [5]. After the seedlings had grown for 30 days, a wounded-root inoculation method was employed. Approximately half of the root system of each plant was wounded using sterilized scissors. Subsequently, 10 mL of the prepared spore suspension was inoculated onto the wounded sites. Control plants were treated with an equal volume of sterile water. Each treatment consisted of 30 biological replicates.

### 2.3. Extraction and Analysis of Metabolites from A. sinensis Rhizosphere Soil, Root Surface, and F. solani

#### 2.3.1. Collection of Rhizosphere Soil

Rhizosphere soil was collected from both diseased and healthy groups 15 days post-inoculation. Six plants were randomly selected from each group. The pots were cut open with scissors, and the plants were carefully removed. Soil loosely adhering to the roots was shaken off, and soil still attached to the roots was gently brushed into a collection container using a sterile brush. Prior to collection, other impurities (e.g., plant debris) on the soil surface were removed. The collected rhizosphere soil was sieved through a 20-mesh sieve to remove visible plant roots, animal residues, and other debris. The soil from each plant was then placed into a 50 mL sterile centrifuge tube, subsequently transferred to a sterile cryovial, immediately placed on dry ice for temporary storage, labeled, and transported to the laboratory.

#### 2.3.2. Collection of Root Surface Samples

Healthy and diseased roots from the above plants were cut into small segments using sterile scissors. The segments were placed separately into 50 mL centrifuge tubes containing 15 mL of sterile phosphate-buffered saline (PBS) solution. The tubes were shaken at 180 rpm at 4 °C for 20 min. After removing the root tissues, they were transferred to a fresh aliquot of sterile PBS solution and subjected to the same shaking conditions for another 20 min. The liquid fractions from the two treatments were combined, mixed thoroughly, and centrifuged at 1000 rpm, 4 °C for 10 min using a high-speed refrigerated centrifuge (Hunan Xiangyi Laboratory Instrument Development Co., Ltd., Changsha, China, Model H-2050R). The supernatant was discarded, and the pellet, representing the root-surface-associated microbial and particulate matter, was collected as the root surface sample.

#### 2.3.3. Qualitative Analysis of Root Secretions

An appropriate amount of sample was accurately weighed into a 2 mL centrifuge tube. Precisely 600.0 µL of a 4 mg/L methanol solution of 2-chloro-L-phenylalanine (internal standard) was added (2-Chloro-L-phenylalanine does not naturally exist in plant and microbial systems and exhibits no interference from endogenous substances in samples. It possesses stable chemical properties and similar physicochemical characteristics to most metabolites, with a steady mass spectrometric response. This compound can effectively correct errors arising from sample pretreatment and instrumental signal drift. Therefore, it was selected as the internal standard for LC-MS metabolomic analysis in this study). The mixture was vortexed for 30 s. A steel bead was added, and the tube was placed into a tissue lyser and ground at 55 Hz for 90 s. The sample was then sonicated at room temperature for 30 min and incubated on ice for 30 min. It was subsequently centrifuged at 12,000 rpm, at 4 °C for 10 min. The supernatant was filtered through a 0.22 µm membrane, and the filtrate was transferred into a vial for liquid chromatography–mass spectrometry (LC-MS) analysis. Six independent biological replicates were performed for both the diseased (DP) and healthy (CK) groups.

#### 2.3.4. Qualitative Analysis of *F. solani* Secretions

An appropriate volume of the cultured and purified *F. solani* suspension was transferred to a sterile centrifuge tube and centrifuged at 5000 rpm, at 4 °C for 5 min to pellet the mycelia and spores. The pellet was washed quickly with pre-chilled PBS solution two to three times. After each wash, the sample was centrifuged at 5000 rpm, 4 °C for 5 min, and the supernatant was completely discarded. The washed cell pellet was collected into a 2 mL cryogenic centrifuge tube, flash-frozen in liquid nitrogen for 15 min, and stored at −80 °C until LC-MS analysis. Six independent biological replicates were performed.

#### 2.3.5. LC-MS Analytical Conditions

Chromatographic conditions: Separation was performed using a Thermo Vanquish ultra-high-performance liquid chromatography (Thermo Fisher Scientific, Bremen, Germany) system equipped with an ACQUITY UPLC^®^ HSS T3 column (2.1 × 100 mm, 1.8 µm; Waters, Milford, MA, USA). The flow rate was 0.3 mL/min, the column temperature was maintained at 40 °C, and the injection volume was 2.0 µL. For positive ion mode, the mobile phase consisted of 0.1% formic acid in acetonitrile (B1) and 0.1% formic acid in water (A1). The gradient elution program was as follows: 0–1 min, 8% B1; 1–8 min, 8% to 98% B1; 8–10 min, 98% B1; 10–10.1 min, 98% to 8% B1; 10.1–12 min, 8% B1. For negative ion mode, the mobile phase consisted of acetonitrile (B2) and 5 mmol/L ammonium formate in water (A2). The gradient elution program was as follows: 0–1 min, 8% B2; 1–8 min, 8% to 98% B2; 8–10 min, 98% B2; 10–10.1 min, 98% to 8% B2; 10.1–12 min, 8% B2 [13].

Mass Spectrometric Conditions: Detection was performed using a Thermo Orbitrap Exploris 120 mass spectrometer (Thermo Fisher Scientific, San Jose, CA, USA) with an electrospray ionization (ESI) source. Data were acquired in both positive and negative ion modes. The spray voltage was set to 3.50 kV for positive mode and −2.50 kV for negative mode. The sheath gas flow was 40 arbitrary units (arb), and the auxiliary gas flow was 10 arb. The capillary temperature was set to 325 °C. Full-scan MS data were acquired at a resolution of 60,000 over a mass range of *m*/*z* 100–1000. Data-dependent MS/MS acquisition was performed using higher-energy collisional dissociation (HCD) with a normalized collision energy of 30%. MS/MS spectra were acquired at a resolution of 15,000 for the top four most intense precursor ions. Dynamic exclusion was enabled to avoid repeated fragmentation of the same ions [14].

### 2.4. Determination of Salicylic Acid (SA) and Jasmonic Acid (JA)

Fresh root samples (1.0 g) from both diseased and healthy *Angelica sinensis* plants were frozen in liquid nitrogen and ground. The homogenate was mixed with 5 mL of 80% methanol, and 10 μL of an internal standard mixture containing 10 ng/mL jasmonic acid (JA) and 60 ng/mL salicylic acid (SA) was added. Ultrasonic extraction was performed for 30 min at 4 °C. Following extraction, the mixture was centrifuged at 12,000× *g* for 10 min. The resulting supernatant was collected, evaporated to dryness, and the residue was redissolved in 2 mL of 80% methanol. After filtration through a 0.22 μm membrane, the concentrations of JA and SA were measured using high-performance liquid chromatography (HPLC) [15] using the internal standard method with Thermo Xcalibur 4.7 software. The calculation formula was: Analyte content = Analyte peak area × (Internal standard concentration/Internal standard peak area). Each treatment consisted of five biological replicates.

### 2.5. Exogenous Application of A. sinensis “Cry for Help” Metabolites

#### 2.5.1. Screening of Mixture Ratios

Due to the varied water solubility of screened potential “cry for help” metabolites from *A. sinensis*, each was prepared into single stock solution with 30% ethanol–water solution. Combined with plant physiological traits, 15 mM was determined as the effective soluble concentration with stable dissolution state. Equal volumes of individual stock solutions were blended to obtain mixed solution, with each metabolite kept at 15 mM. For soil pretreatment, 1 mL of this master mixture was diluted with deionized water to a final volume of 100 mL to prepare the spraying solution. Then, 5 mL of this working solution was evenly sprayed onto and mixed with 100 g of sterilized soil. This application was repeated every 3–4 days for a total of 8 times. Following the final application, the soil was aged for 7 days. The aged treated soil was mixed with sterilized blank soil at mass ratios of 1:1, 1:2, and 1:3. These dilution gradients were screened via preliminary assays to match natural metabolite concentration ranges under field conditions (this study aimed to verify the biological effectiveness of target metabolites; hence, three distinctly different ratios were selected for investigation, which did not represent the optimal treatment concentrations). All soil mixtures were separately placed into sterilized pots, with five replicates per group. *A. sinensis* seedlings were transplanted and grown for 15 days, after which the disease model was established according to the method described in Section 2.2. After a total growth period of 30 days post-transplanting, samples were collected to measure various growth and disease resistance indicators. By comparing the differences between the various treatment groups and the control group, the optimal soil treatment concentration was screened.

#### 2.5.2. Screening of Key Metabolites

Based on the aforementioned results, each candidate metabolite was individually mixed with sterilized soil. These mixtures were then combined with regular soil at the optimal ratio determined previously. Control groups (healthy and diseased) were established in parallel. The disease model was established using the same methodology. After 30 days of growth, *A. sinensis* seedlings were sampled for the measurement of growth indices, disease index, and enzyme activities. Each treatment group consisted of five biological replicates.

#### 2.5.3. Measurement of Indices

Seedling height and root length were measured using a ruler. Root crown diameter was measured with a vernier caliper. Aboveground and belowground biomass were determined using a 0.0001 g precision electronic balance. The disease index was assessed on a 0 to 4 scale: Grade 0: healthy roots with no visible lesions; Grade 1: lesion area covering < 5% of the total root surface area; Grade 2: lesion area covering 5% to 10% of the total root surface area; Grade 3: lesion area covering 10% to 15% of the total root surface area; and Grade 4: lesion area covering 15% to 20% of the total root surface area. After recording the disease grade for each plant, the disease index was calculated using the following formula: Disease Index = [Σ (Disease grade × Number of plants in that grade)/(Highest disease grade × Total number of plants)] ×100. The activities of lipoxygenase (LOX), phenylalanine ammonia-lyase (PAL), polyphenol oxidase (PPO), and peroxidase (POD) in root tissues were determined using enzyme-linked immunosorbent assay (ELISA).

### 2.6. Rhizosphere Soil Microbiome Extraction and Sequencing

#### 2.6.1. Rhizosphere Soil Collection

Rhizosphere soil was collected from *A. sinensis* plants in each treatment group after 30 days of growth. To maintain aseptic conditions, face masks and disposable sterile gloves were worn during sampling, with gloves changed between different treatment groups. Six plants were randomly selected from each group. Pots were cut open using scissors, and the entire plant was carefully removed. The bulk soil was gently shaken off the roots, and the soil closely adhering to the roots was subsequently collected by gently brushing off using a sterile brush. The rhizosphere soil from each individual plant was placed into a separate sterile cryogenic vial and immediately placed on dry ice for temporary storage prior to microbial analysis.

#### 2.6.2. Soil DNA Extraction and Sequencing

Total genomic DNA was extracted from soil samples using the DNeasy PowerSoil Kit (Qiagen, Hilden, Germany). The V3-V4 hypervariable region of the bacterial 16S ribosomal DNA (rDNA) was amplified by PCR using the primer pair 341F (5′-CCTACGGGNGGCWGCAG-3′) and 785R (5′-GACTACHVGGGTATCTAATCC-3′). The fungal internal transcribed spacer (ITS) region was amplified using the primer pair ITS1-F (5′-CTTGGTCATTTAGAGGAAGTAA-3′) and ITS2-R (5′-GCTGCGTTCTTCATCGATGC-3′). The PCR reaction was performed in a 30.0 μL volume containing 15.0 μL of 2× Gflex PCR Buffer, 0.6 μL of Tks Gflex DNA Polymerase, 1.0 μL of DNA template, 1.0 μL each of forward and reverse primers (5 pmol/μL), and ddH_2_O to a final volume of 30.0 μL. The thermal cycling program was as follows: initial denaturation at 96 °C for 5 min; 40 cycles of denaturation at 96 °C for 45 s, annealing at 56 °C for 45 s, and extension at 72 °C for 45 s; followed by a final extension at 72 °C for 5 min. The purity of the PCR products was assessed by 1% agarose gel electrophoresis. Qualified amplicons were sent to Chongqing Xinghewan Biotechnology Co., Ltd. (Chongqing, China) for sequencing. If no band was visible for the negative control, a second round of PCR amplification was performed. The primary PCR products were purified using the AMPure XP Kit (Beckman Coulter, Brea, CA, USA), diluted to 50 ng/μL, and used as the template for a second PCR under the same conditions but for only 8 cycles. Subsequently, 5.0 μL of the purified secondary PCR product was analyzed by 1% agarose gel electrophoresis to verify the presence and specificity of the band, while 1.0 μL was used to determine DNA concentration using a Nanodrop spectrophotometer (Thermo Fisher Scientific, Wilmington, DE, USA). Six independent biological replicates were performed for both the diseased and healthy groups. Upon passing quality control, the samples were sent to Chongqing Xinghewan Biotechnology Co., Ltd. for paired-end sequencing (2 × 250 bp) on an Illumina HiSeq PE250 platform (Illumina, Inc., San Diego, CA, USA) for both 16S rDNA and ITS rDNA.

### 2.7. Statistical Analysis

#### 2.7.1. Metabolomics Data Analysis

Partial least squares-discriminant analysis (PLS-DA) was performed on the data matrix using the ropls package in R (v4.2.0). Differential metabolites were screened based on a variable importance in projection (VIP) value greater than 1.0 derived from the PLS-DA model and a *p*-value less than 0.05 from a two-tailed Student’s *t*-test. The fold change (FC) was calculated as the binary logarithm (log_2_) of the ratio of the average normalized peak intensities between two groups. Metabolites with an absolute log_2_FC greater than 1 were considered significant. Volcano plots and heatmaps for inter-group comparisons were generated using the online platform MetaboAnalyst (v 6.0).

#### 2.7.2. Metabolite Identification and Quality Control

Metabolite annotation was performed by matching precursor *m*/*z*, adduct ions, and MS/MS fragment spectra against HMDB, MassBank, KEGG, LipidMaps, mzCloud, and an in house database. Quality control (QC) samples were prepared by pooling equal aliquots of all samples and injected at regular intervals. Systematic errors were corrected using support vector regression (SVR) based on QC sample data.

#### 2.7.3. Microbiome Data Analysis

Raw paired-end sequencing reads were merged, quality-filtered, and chimera-checked to obtain high-quality sequences. These optimized sequences were then clustered into amplicon sequence variants (ASVs) at 100% similarity using Vsearch (version 2.27.1). Representative 16S rDNA ASV sequences were taxonomically annotated by alignment against the Greengenes database, while representative ITS sequences were annotated using the UNITE database. Alpha diversity analysis was conducted using QIIME (version 1.9.1). Principal component analysis (PCA) was performed using the vegan package (version 2.5.6) in R.

Differences in growth indices, disease index, enzyme activities, and microbial metrics among groups were assessed for significance using one-way analysis of variance (ANOVA) followed by Duncan’s multiple range test in SPSS software (version 26.0). A *p*-value of less than 0.05 was considered statistically significant.

## 3. Results

### 3.1. Metabolites in F. solani Culture Filtrate

**Figure 1:**
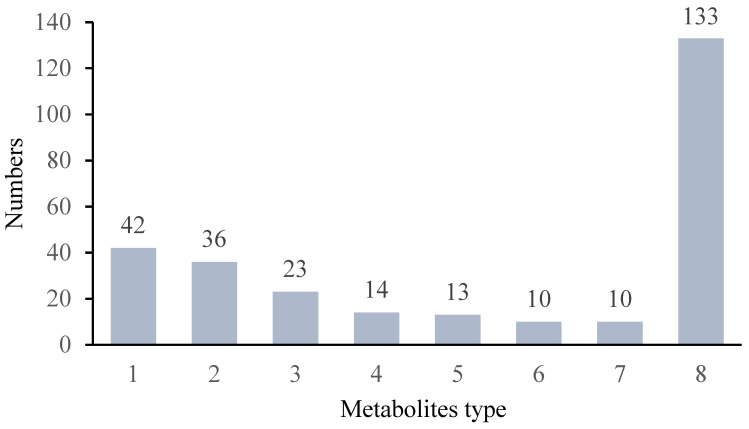
Metabolite Composition of *F. solani.* Metabolites types: 1: Carboxylic acids and derivatives; 2: Fatty acyls; 3: Organooxygen compounds; 4: Benzene and substituted derivatives; 5: Steroids and steroid derivatives; 6: Phenols; 7: Pyridines and derivatives; 8: Others.

LC-MS analysis detected and identified a total of 281 metabolites in the *F. solani* culture filtrate. The major classes included carboxylic acids and derivatives (14.9%), fatty acyls (12.8%), organooxygen compounds (8.2%), benzenes and substituted derivatives (4.9%), steroids and steroid derivatives (4.6%), phenols (3.6%), and pyridines and derivatives (3.6%), among others (Figure 1). The ten most abundant metabolites were erucic acid, oleic acid, glycerophosphocholine, palmitic acid, aminocaproic acid, caproic acid, 3-hydroxybenzyl alcohol glucoside, octadecanamide, arachidic acid, and 4-hydroxycinnamic acid. Notably, fatty acyls constituted over half of these top metabolites.

### 3.2. Differential Metabolites in the Root Zone of Healthy and Diseased A. sinensis

A total of 358 metabolites were detected and identified across all soil samples. The major classes included fatty acyls (15.1%), carboxylic acids and derivatives (11.6%), organooxygen compounds (6.9%), benzenes and substituted derivatives (6.5%), and steroids and steroid derivatives (5.8%). In all root samples, 604 metabolites were detected and identified. Predominant classes comprised carboxylic acids and derivatives (16.4%), amino acids, peptides, and analogues (11.6%), organooxygen compounds (7.8%), and steroids and steroid derivatives (4.8%).

**Figure 2:**
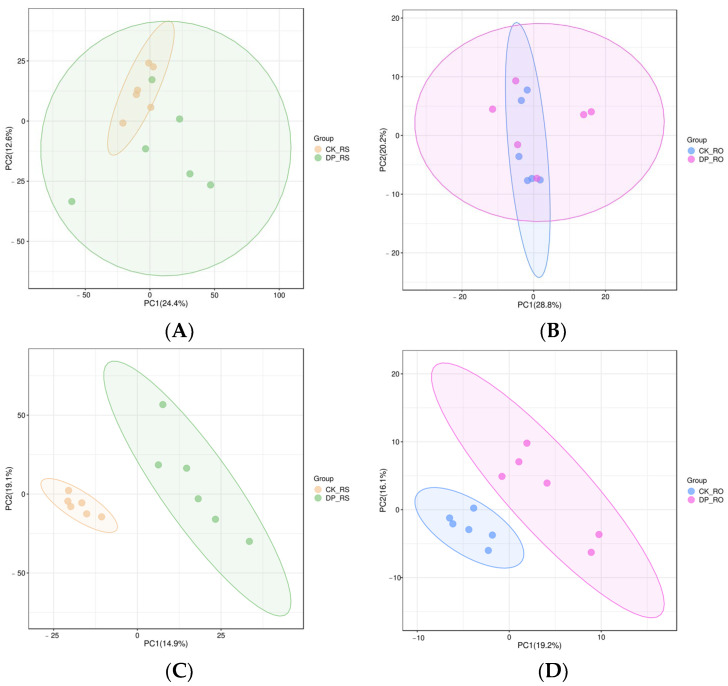
PCA score plots of metabolic profiles in rhizosphere soil (**A**) and roots (**B**) of *Angelica sinensis*. and PLS-DA score plots of the metabolite profiles from the rhizosphere soil (**C**) and roots (**D**) of *A. sinensis*.

The PCA score plots (Figure 2) show that both rhizosphere soil and root metabolite samples were within the 95% confidence interval. Under *F. solani* stress, the metabolite composition of rhizosphere soil in the healthy group and the diseased group did not exhibit a complete separation trend along the coordinate axes and showed low discreteness. Samples from diseased rhizosphere soil were clearly separated with relatively large characteristic differences. The horizontal and vertical coordinates of its PCA score plot accounted for 24.4% and 12.6%, respectively. The first principal component (PC1) and the second principal component (PC2) explained 37.0% of the total diversity of root exudates, with PC1 making a prominent contribution. Diseased root samples showed obvious separation, and the degree of dispersion was significantly higher than that of rhizosphere soil. Root sample points were also separated, but the difference was significantly lower than that in rhizosphere soil. The horizontal and vertical coordinates of its PCA score plot accounted for 28.8% and 20.2%, respectively. PC1 and PC2 explained 49.0% of the total diversity of root exudates, with both principal components making substantial contributions. These results indicate that *F. solani* stress has a considerable impact on the metabolite composition of *A. sinensis*.

The PLS-DA score plots revealed a clear separation in metabolite profiles between the healthy and diseased *A. sinensis* groups in both the rhizosphere soil (Figure 2C) and the roots (Figure 2D) following *F. solani* inoculation. For the rhizosphere soil, the first two principal components in the PLS-DA model explained 14.9% and 19.1% of the total variance, respectively. For the root samples, the corresponding values were 19.2% and 16.1%. These results indicate that *F. solani* infection significantly altered the metabolite composition in both the rhizosphere soil and the roots of *A. sinensis*. Although these results indicate that *F. solani* infection altered metabolite compositions, the relatively low cumulative explained variance suggests that the detected metabolites represent only part of a more complex situation; other unmeasured metabolites, microbial factors, or environmental variables may also contribute.

**Figure 3:**
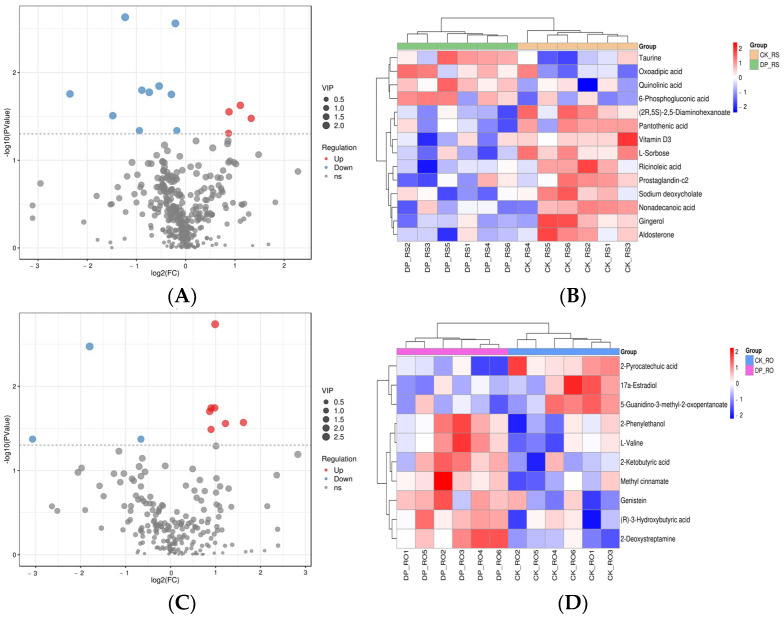
Differential metabolites in the rhizosphere soil and roots of diseased (DP) versus healthy (CK) *A. sinensis*. (**A**) Volcano plot of differential metabolites between DP-RS and CK-RS groups in rhizosphere soil; (**B**) Clustering heatmap of key differential metabolites in rhizosphere soil; (**C**) Volcano plot of differential metabolites between DP-RO and CK-RO groups in roots; (**D**) Clustering heatmap of key differential metabolites in roots.

As shown in Figure 3, in the rhizosphere soil, the diseased group exhibited significant upregulation of 4 metabolites and downregulation of 10 metabolites compared to the healthy group (Figure 3A). The upregulated metabolites were taurine, oxoadipic acid, quinolinic acid, and 6-phosphogluconic acid (Figure 3B). In the roots, the diseased group showed significant upregulation of 7 metabolites and downregulation of 3 metabolites compared to the healthy group (Figure 3C). The upregulated metabolites were 2-phenylethanol, L-valine, 2-ketobutyric acid, methyl cinnamate, genistein, (R)-3-hydroxybutyric acid, 2-deoxystreptamine acid (Figure 3D). Comparison with the *F. solani* metabolome revealed that among the 11 metabolites significantly upregulated in the rhizosphere soil and roots of diseased *Angelica sinensis*, 7 were unique metabolites: taurine, oxoadipic acid, quinolinic acid, 6-phosphogluconic acid, methyl cinnamate, and 2-phenylethanol, (R)-3-hydroxybutyric acid. According to the “cry for help” theory, these metabolites may represent potential “cry for help” signals from *A. sinensis*.

### 3.3. Differences in Key Defense-Related Hormones Between Healthy and Diseased A. sinensis

**Figure 4:**
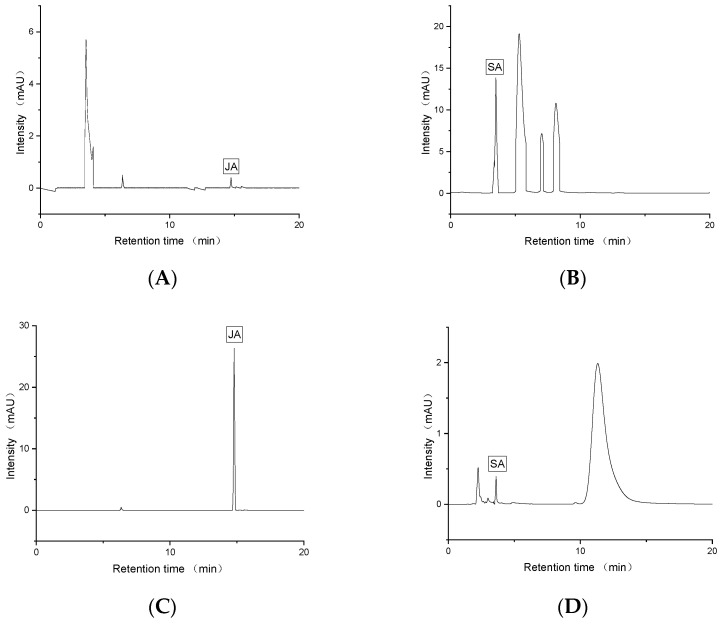
Changes in the levels of key defense-related hormones in healthy and diseased *A. sinensis*. (**A**,**B**) show the levels of JA and SA in healthy plants, while (**C**,**D**) represent the JA and SA levels in diseased plants.

As shown in Figure 4, significant differences were observed between healthy and diseased *A. sinensis* plants regarding levels of the key defense-related hormones, JA and SA. Compared to the healthy group, the diseased group exhibited a significant increase in JA concentration (*p* < 0.05), while the SA concentration decreased significantly (*p* < 0.05).

### 3.4. Potential of the A. sinensis “Cry for Help” Metabolite Mixture to Mitigate Root Rot

**Figure 5:**
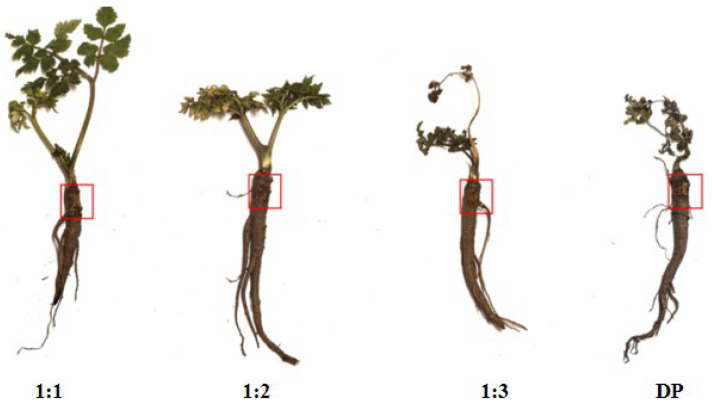
Growth trends of *A. sinensis* in mixed soils with different proportions. The red box indicates the typical root rot lesion on the root crown of *A. sinensis*.

As shown in Table 1 and Figure 5, the root length, belowground biomass, root diameter, and aboveground biomass of *A. sinensis* seedlings in the 1:1 mixture treatment group (treated soil to regular soil) were significantly higher than those in the diseased control group and the other treatment groups (*p* < 0.001). This result demonstrates not only the potential of the metabolite mixture to mitigate root rot but also indicates that the 1:1 dilution ratio exerted a more pronounced alleviating effect. Therefore, the 1:1 ratio was selected for subsequent experiments aimed at screening the active “cry for help” compounds.

### 3.5. Effects of Different “Cry for Help” Metabolites on Plant Growth and Defense-Related Enzyme Activities in A. sinensis

To further screen for key metabolites with “cry for help” capacity, taurine (T), oxoadipic acid (OXY), 6-phosphogluconic acid (P-6), quinolinic acid (Q), methyl cinnamate (EMC), (R)-3-hydroxybutyric acid (R-3HB), and 2-phenylethanol (B-2) were individually mixed with sterilized soil. These treated soils were then mixed with regular soil at a 1:1 ratio. A healthy control (CK) group and a diseased (DP) group were established simultaneously. Phenotypic comparative analysis was subsequently performed on *Angelica sinensis* plants under these nine treatments.

As shown in Table 2, The plant height, root length, above-ground biomass, and below-ground biomass of the DP group were significantly lower than those of the CK group. Among the remaining treatments, the T, EMC, R3HB, and B2 groups did not show a declining trend in growth indicators such as plant height, root length, below-ground biomass, and rhizome diameter compared with the CK group. However, the growth performance of *A. sinensis* in the T and EMC groups was significantly better than that in the R3HB and B2 groups. In contrast, most indicators of the Q, OXY, and P6 groups were only slightly better than those of the DP group.

Regarding disease control, the disease indices of the T, EMC, R3HB, and B2 groups were significantly lower than that of the DP group, with the T and EMC groups showing the most pronounced reduction.

Defensive enzyme activities were assayed in root samples of *A. sinensis* from the nine treatment groups. As shown in Table 3, the activities of peroxidase (POD), phenylalanine ammonia-lyase (PAL), lipoxygenase (LOX), and polyphenol oxidase (PPO) in the DP group were higher than those in the CK group, indicating that disease infection increased the secretion of defensive enzymes and enhanced the disease resistance of *A. sinensis*. Among the other treatment groups, the EMC group showed slightly higher enzyme activities than the DP group, suggesting that EMC may have greater potential in promoting the secretion of defensive enzymes in plants. Although the enzyme activities in the T group were lower than those in the EMC group, they still exhibited a modest enhancing effect compared to the DP group. The B-2 group displayed enzyme activities essentially similar to those of the DP group. Both the T and B-2 groups may contribute to antibacterial activity or recruitment of beneficial microorganisms. The R-3HB and OXY groups showed slightly higher enzyme activities than the CK group, while the Q group and most enzymes in the P-6 group had activities comparable to those of the CK group. However, the phenylalanine ammonia-lyase (PAL) activity in the P-6 group was significantly elevated and higher than that in all other groups. These results indicate that different treatments vary in their potential to activate the active defense response of *A. sinensis*, and the same treatment can also differentially influence the activities of distinct defensive enzymes.

### 3.6. Effects of Different”Cry for Help”Metabolites on the Rhizosphere Microorganisms of A. sinensis

**Figure 6:**
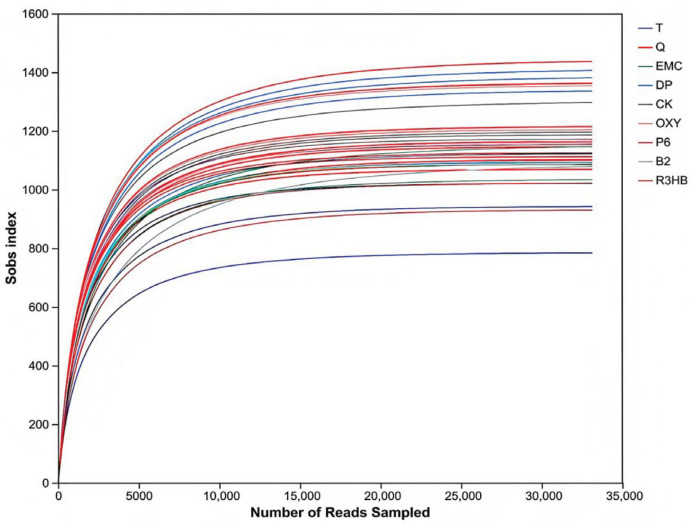
Rarefaction curves of bacteria in rhizosphere soil samples of *Angelica sinensis*.

As shown in Figure 6, alpha diversity analysis of the bacterial community in the rhizosphere soil of *A. sinensis* revealed that, after quality control, the clean tags ranged from 67,939–79,630 across samples. After chimera removal, the number of valid tags finally used for analysis ranged from 67,931–791,592. The rarefaction curves corresponding to the rhizosphere soil samples of both healthy and diseased *A. sinensis* plants all tended to plateau, and the sequencing coverage of the bacterial library exceeded 92.1%. These results indicate that the sequencing depth was sufficient, the community structure exhibited a high level of confidence, and the data could reliably reflect the actual bacterial community structure of the samples.

As shown in Table 4, ANOVA results revealed significant differences among treatments in the soil bacterial ACE, Chao1, Shannon, and Simpson indices (*p* < 0.01). Pairwise comparisons further showed that the species richness (ACE and Chao1) in the diseased (DP) group was significantly higher than that in the healthy control (CK) group (*p* < 0.05), whereas no significant differences were observed in community diversity or dominance (Shannon and Simpson indices) between the two groups (*p* > 0.05). These results indicate that under *F. solani* stress, both the richness and diversity of the rhizosphere bacterial community of *A. sinensis* undergo significant changes, and pathogen invasion disrupts the overall balance of the rhizosphere microbial community of healthy plants. Furthermore, the exogenously added metabolites regulated the rhizosphere bacterial community through different mechanisms, thereby further optimizing the compositional structure of the rhizosphere microbiome of *A. sinensis*.

**Figure 7:**
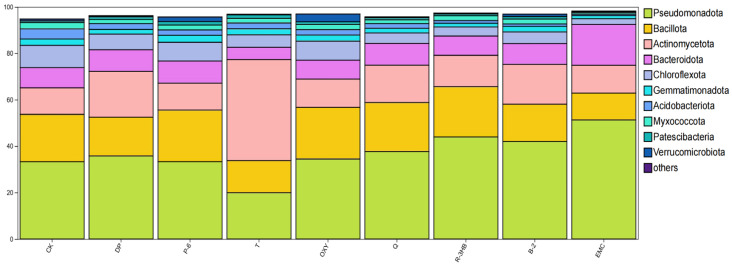
Composition of the rhizosphere soil bacterial community in *A. sinensis* under different treatments at the phylum level.

Analysis of the rhizosphere soil bacterial composition in *A. sinensis* across nine treatment groups revealed that the dominant bacterial composition at the phylum level was largely consistent. Ten phyla—*Pseudomonadota*, *Bacillota*, *Actinomycetota*, *Chloroflexota*, *Bacteroidota*, *Gemmatimonadota*, *Myxococcota*, *Acidobacteriota*, *Patescibacteria*, and *Verrucomicrobiota*—collectively accounted for an average relative abundance exceeding 96.71% of the total community, although inter-group differences in relative abundance were observed. As shown in Figure 7, the relative abundance of *Actinomycetota* in the T group (43.5%) was significantly higher (*p* < 0.05) than that in the CK (11.4%) and DP (19.7%) groups. Similarly, the EMC group exhibited significantly increased relative abundances of *Bacteroidota* (17.6%) and *Pseudomonadota* (51.2%) compared to the CK group (8.7% and 33.3%, respectively) and the DP group (9.3% and 35.8%, respectively). No other significant differences were detected among the dominant bacterial phyla in the remaining treatment groups.

As shown in Table 5, significant differences were observed in the dominant bacterial genera within the rhizosphere soil of *A. sinensis* across the nine treatment groups. The predominant genera mainly included *Rhizobacter*, *Sphingomonas*, *Cellvibrio*, *Bacillus*, *Devosia*, unclassified *Rhizobiaceae*, *Luteimonas*, *JG30-KF-CM45*, *Rummeliibacillus*, *Methylocaldum*, *Pseudomonas*, *Lysobacter*, *Planococcus*, *Planifilum*, *Paeniglutamicibacter*, *Azotobacter*, *MND1*, and unclassified *Micrococcaceae*, totaling 18 bacterial genera. The distribution patterns of these dominant genera varied among the different groups.

In the CK group, *Rhizobacter* (7.2%), *Sphingomonas* (6.5%), and *Pseudomonas* (3.1%) exhibited the highest relative abundances, collectively accounting for 16.8% and forming the core taxa of the rhizobacterial community in this group. Additionally, the CK group contained stress-tolerant related genera such as *Methylocaldum* (2.4%) and *Rummeliibacillus* (2.3%). Both the T and EMC groups exhibited pronounced enrichment of specific functional bacterial taxa. In the T group, the relative abundance of *Paeniglutamicibacter* reached 22.1%, significantly exceeding that in all other treatment groups, followed by unclassified *Micrococcaceae* (5.9%), with these two genera together comprising 28.0%. In the EMC group, the relative abundance of *Azotobacter* was as high as 21.4%, and notably, *Azotobacter* was not detected among the top 10 dominant genera in any of the other eight treatment groups. The secondary dominant genus in the EMC group was *Bacillus* (2.7%), with these two genera collectively accounting for 24.1%. The other treatment groups generally showed no absolutely dominant bacterial genus, displaying a relatively balanced distribution of genus-level abundances. Furthermore, *Pseudomonas*, *Devosia*, and unclassified *Rhizobiaceae* were consistently ranked among the top 10 dominant genera in most treatment groups, indicating they are core shared dominant genera in the rhizosphere of *A. sinensis*.

**Figure 8:**
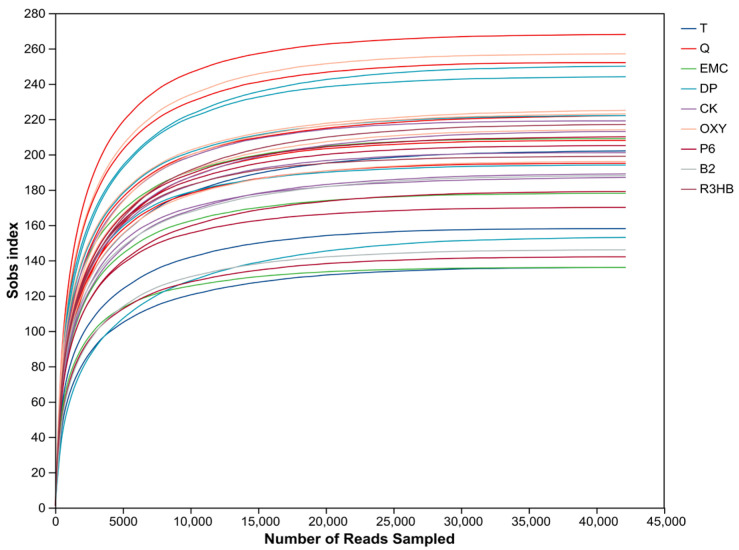
Fungal rarefaction curves of rhizosphere soil samples of *Angelica sinensis*.

The rarefaction curves corresponding to the fungal fraction of rhizosphere soil samples from both healthy and diseased *A. sinensis* plants all tended to plateau, and the library sequencing coverage exceeded 99.9%. As shown in Figure 8, these results indicate that the sequencing depth was sufficient, the community structure exhibited a high level of confidence, and the data could reliably reflect the actual fungal community structure of the samples.

As shown in Table 6, ANOVA results revealed significant differences in fungal ACE, Chao1, Shannon, and Simpson indices of rhizosphere soil among all treatment groups (*p* < 0.05). Multiple comparison results showed that under *F. solani* stress, fungal species richness, as reflected by the ACE and Chao1 indices, in the diseased (DP) group was numerically higher than that in the healthy control (CK), though the difference was not statistically significant (*p* > 0.05). The DP group exhibited a much larger standard deviation across all alpha diversity indices, demonstrating that pathogen infection led to greater heterogeneity and poorer stability of the rhizosphere fungal community. No significant difference in Shannon diversity was detected between DP and CK, whereas DP had a significantly higher Simpson index, which implied reduced community evenness under pathogenic stress. The variations in microbial diversity across different treatments confirmed that exogenous metabolites could effectively regulate the rhizosphere fungal community structure of *A. sinensis*. Treatments EMC and T markedly decreased fungal species richness, while other functional compounds optimized the rhizosphere fungal microecology by modulating community diversity and evenness.

**Figure 9:**
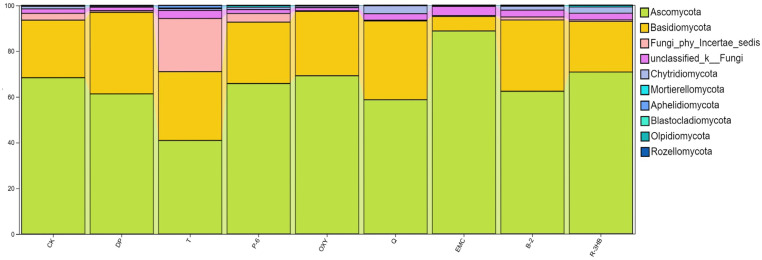
Composition of the rhizosphere soil fungal community in *A. sinensis* under different treatments at the phylum level.

Analysis of the fungal composition in the rhizosphere soil of *A. sinensis* across nine treatment groups revealed that the dominant fungal composition at the phylum level was fundamentally consistent. The relative abundances of ten phyla—*Ascomycota*, *Basidiomycota*, *Chytridiomycota*, *phy_Incertae_sedis_Fungi*, *Fungi*_unclassified, *Mortierellomycota*, *Olpidiomycota*, *Aphelidiomycota*, *Zoopagomycota*, and *Mu*coromycota—collectively accounted for an average of over 99.79% of the total community, although inter-group differences in relative abundances were observed. As shown in Figure 9, the relative abundance of *Ascomycota* in the T group (42.3%) was significantly lower than that in the CK (75.4%) and DP (70.4%) groups, while the relative abundance of *Chytridiomycota* (23.3%) was significantly higher compared to the CK (0.2%) and DP (1.4%) groups. The EMC group exhibited a significantly higher relative abundance of *Ascomycota* (86.86%) than the other groups. The relative abundance of the *Fungi*_unclassified phylum was significantly higher in the CK (1.2%), Q (3.5%), R-3HB (2.7%), and B-2 (1.7%) groups compared to the DP (0.4%), T (0.8%), EMC (0.4%), P-6 (1.0%), and OXY (0.7%) groups. No significant changes were observed for the other phyla among the groups.

As shown in Table 7, significant differences were also observed in the dominant fungal genera within the rhizosphere soil of *A. sinensis* among the nine treatments at the genus level. The dominant fungal genera in the CK group included *Coprinus*, *Schizothecium*, *Thermomyces*, *Aspergillus*, *Iodophanus*, *Stachybotrys*, *Scedosporium*, *Peziza*, *Gibberella*, and *Xanthothecium*. In contrast, the dominant genera in the DP group and other treatment groups primarily consisted of *Coprinus*, *Plectosphaerella*, *Fusarium*, unclassified *Sordariaceae*, *Olpidiaster*, *Gaertneriomyces*, *Rhizophlyctis*, unclassified genera, unclassified *Lasiosphaeriaceae*, *Thermomyces*, *Iodophanus*, unclassified *Hypocreales*, *Fungi_gen_Incertae_sedis*, *Alternaria*, and *Coprinellus*. This indicates that *F. solani* infection significantly altered the fungal community composition in the rhizosphere soil of *A. sinensis*, although *Coprinus* remained the core dominant fungal genus common to all groups.The results showed that the relative abundance of *Fusarium* decreased in all treatment groups compared to the DP group. The most pronounced decreasing trends were observed in the T group (2.7%) and the EMC group (2.0%), which were substantially lower than that in the DP group (10.1%). Although the majority of dominant fungal genera were consistent between the treatment groups and the DP group, inter-group differences in relative abundance were still evident. Notably, the relative abundance of *Schizothecium* in the EMC group (38.8%) was significantly higher than that in the DP group (4.2%).

## 4. Discussion

### 4.1. Metabolic Characteristics of F. solani

LC-MS analysis of the metabolites in the *F. solani* culture filtrate revealed that carboxylic acids and derivatives, along with fatty acyls, were the most abundant substance classes, indicating highly active basal energy metabolism and lipid metabolism in the fungal cells [16]. Among the high-abundance metabolites of *F. solani*, erucic acid, oleic acid, palmitic acid, and arachidic acid are all long-chain fatty acids. Oleic acid and palmitic acid have been confirmed as core components of fungal cell membrane phospholipids [17]. It has been reported that fatty acyls, organic acids, and phenolic compounds are commonly produced by various *Fusarium* species, including *F. oxysporum* and *F. graminearum* [18], indicating conserved core metabolic traits within the genus. A direct comparative metabolomic study between *F. solani* and *F. oxysporum* further revealed species-specific differences, such as a higher abundance of saturated fatty acids in *F. solani* [19]. In agreement with these findings, our analysis showed a high abundance of oleic acid, and palmitic acid in *F. solani* culture filtrates, suggesting that these specific fatty acyls may contribute to a species-specific metabolic signature of *F. solani.* However, erucic acid is commonly found in seeds of some oil crops [20] and is generally present at low levels in fungi. Its enrichment in this *F. solani* strain may be related to specific metabolic pathways or culture conditions. Furthermore, high-concentration metabolites of *F. solani* also include short-chain fatty acids such as hexanoic acid and aminohexanoic acid. It has been reported that hexanoic acid may inhibit plant growth and accelerate necrosis of plant tissue cells by reducing the pH of the rhizosphere microenvironment and increasing the production of toxins like deoxynivalenol (DON) and fumonisins, thereby creating conditions for hyphal invasion [21]. Concurrently, 4-Hydroxycinnamic acid, as a precursor for lignin synthesis [22], may induce the fungal cells to synthesize lignin-degrading enzymes, thereby degrading the cell wall components of *A. sinensis* roots and facilitating the penetration of hyphae through the host cell wall. Furthermore, under weakly acidic conditions, 4-hydroxycinnamic acid can also inhibit the activity of polyphenol oxidase (an important plant defense-related enzyme). This explains why *Fusarium solani* secretes acidic substances to create an acidic microenvironment. Reports indicate that 4-hydroxycinnamic acid is a major driver for the enrichment of *Pseudomonas* bacteria [23]. It is possible that *F. solani* utilizes this compound to drive co-infection of *A. sinensis* by pathogenic *pseudomonads*.

### 4.2. Impact of F. solani on the Metabolome of the A. sinensis Root Zone

The results of the partial least squares-discriminant analysis statistically confirmed the strong disruptive effect of *F. solani* on the metabolomes of both the rhizosphere soil and the roots of *A. sinensis*. The distinct separation between the CK and DP groups in the coordinate space indicates that pathogen invasion induced a directional shift in the metabolic network of the *A. sinensis* root zone system. Similar phenomena have been reported in studies on Zanthoxylum bungeanum [24], soybean [25], and wheat [26].Carboxylic acids and derivatives (16.4%) were the dominant class of metabolites in the *A. sinensis* roots. These compounds are involved not only in energy provision for both primary and secondary plant metabolism [27] but are also closely associated with plant stress resistance [28,29]. In contrast, fatty acyls (15.1%) constituted the predominant metabolite class in the rhizosphere soil. These substances primarily originate from root exudates [30], degradation products of microbial cell membranes [31], and the decomposition of soil organic matter [32]. They play crucial roles in cell membranes, participating in cellular signaling and energy metabolism [33]. Their high abundance reflects a high level of activity within the microbial community of the *A. sinensis* rhizosphere soil.

The alterations in *A. sinensis* metabolites induced by *F. solani* represent an active response of the plant to biotic stress. According to the plant “cry for help” theory, when plants are infected by pathogens, they secrete metabolites with direct antimicrobial effects, those that enhance plant resistance, and those that recruit beneficial microorganisms to aid in their defense [34]. The results indicate that under *F. solani* stress, 11 metabolites were significantly upregulated in both the rhizosphere soil and roots of *A. sinensis*. Comparative analysis with the *F. solani* metabolome revealed that taurine, oxoadipic acid, gluconic acid 6-phosphate, quinolinic acid, methyl cinnamate, R-3-hydroxybutyric acid, and 2-phenylethanol were specifically upregulated in *A. sinensis* under pathogen stress.

Taurine, an amino acid ubiquitously distributed in mammalian body fluids and tissues, plays roles in regulating ion homeostasis, osmoregulation, and protein phosphorylation in mammalian cells, protecting them from various diseases affecting visceral organ systems. Its content in plants is generally low [35]. Recent studies suggest that taurine not only regulates radical scavenging, secondary metabolism, and ion homeostasis in plants under stress, significantly improving their vitality in terms of growth, photosynthetic pigments, and nutrient uptake [36], but also mediates plant responses to both abiotic and biotic stresses [37]. However, the mechanisms by which taurine regulates plant defense against external stress remain unclear.

Studies have shown that methyl cinnamate possesses significant antimicrobial properties [38]. Furthermore, derivatives of methyl cinnamate have been found to inhibit Phytophthora nicotianae and recruit beneficial microorganisms [39].

JA and SA are two core hormones involved in plant responses to biotic stress, typically regulating plant disease resistance through an antagonistic relationship [40]. Studies indicate that in *A. sinensis* plants infected by *F. solani*, the levels of JA and SA also exhibit this inversely correlated, antagonistic pattern: JA levels increase significantly, while SA levels decrease.

This reciprocal change suggests a specific functional shift in the defense strategy of *A. sinensis* against *F. solani*. SA is generally associated with resistance against biotrophic and hemibiotrophic pathogens by activating systemic acquired resistance (SAR) and pathogenesis-related (PR) gene expression [41,42]. In contrast, JA predominantly mediates defense against necrotrophic pathogens and herbivorous insects by inducing the production of antimicrobial compounds such as defensins and phytoalexins [43,44]. *F. solani* is a soil-borne necrotrophic or hemibiotrophic fungus that kills host cells to obtain nutrients. *F. solani* is a soil-borne necrotrophic/hemibiotrophic pathogen that obtains nutrients by killing host cells. The elevated JA level in infected roots of *A. sinensis* indicates an active defense response tailored to this type of pathogen. The concomitant decrease in SA may result from either pathogen-mediated suppression of the SA pathway or the natural antagonistic crosstalk between JA and SA signaling, which allows plants to prioritize one defense route to optimize resource allocation [45]. Collectively, these findings indicate that *A. sinensis* activates a JA-centered defense module upon *F. solani* infection, and the downregulation of SA may be a consequence of hormonal antagonism rather than a passive collapse of defense.

### 4.3. Holistic Regulation of A. sinensis by “Cry for Help” Metabolites

#### 4.3.1. Regulation of Growth Phenotype

From the comparison of growth indicators among the nine treatment groups, it can be seen that the protective effects of different “cry for help” compounds on the growth of *A. sinensis* can be broadly categorized into three types: highly protective (T, EMC), moderately protective (Q, B-2, P-6), and weakly protective (OXY, R-3HB). This variation is likely related to the intrinsic physiological functions of the compounds and their compatibility with the stress environment. The highly protective groups supplemented with taurine (T) and methyl cinnamate (EMC) not only maintained normal growth of *A. sinensis* but also effectively suppressed the disease caused by *F. solani*. Existing research suggests that taurine may be a resistance-related (RR) metabolite against Fusarium root rot [46]. This is likely because taurine, acting as a typical osmolyte and antioxidant, can mitigate oxidative damage caused by the pathogen by scavenging reactive oxygen species (ROS), while simultaneously maintaining cellular osmotic potential and safeguarding root absorption functions [47]. Methyl cinnamate, as an antimicrobial substance, may directly inhibit the mycelial growth and spore germination of *F. solani,* reducing disease pressure at the source [48].

The moderately protective compounds—quinolinic acid, 2-phenylethanol, and 6-phosphogluconic acid—function more in terms of basic physiological maintenance, signaling, and moderate antimicrobial efficacy. This is likely because quinolinic acid exhibits antifungal activity and demonstrates certain antioxidant functions in the presence of elements such as iron and manganese [49]; however, it is effective only at extremely low concentrations. At elevated concentrations, it may even promote *F. solani* growth and possesses inherent phytotoxicity. 2-Phenylethanol acts as a plant signaling molecule and, when applied alone, shows some inhibitory effect against the pathogen. 6-Phosphogluconic acid, on the other hand, supports the growth and development of *A. sinensis* by enhancing the activity of its defensive enzymes.

The weakly protective compounds, oxoadipic acid and R-3-hydroxybutyric acid, are primarily metabolic intermediates. Oxoadipic acid is mainly involved in the interplay of central carbon metabolism, amino acid metabolism, and fatty acid metabolism [50]. R-3-hydroxybutyric acid, as a ketone body metabolite, can regulate the life processes of *A. sinensis* under stress by serving both as a metabolite and a signaling molecule. It provides additional energy to meet basic growth requirements and can alter gene expression during plant stress responses; however, it lacks strong antimicrobial or antioxidant capacity [51]. When present alone, these two metabolites exhibit limited physiological activity and are unable to effectively alleviate disease pressure.

#### 4.3.2. Regulation of Defense-Related Enzyme Activities

The results showed that the activities of defense-related enzymes, including POD, PAL, LOX, and PPO, in the DP group were significantly higher than those in the CK group. This indicates that *A. sinensis* actively upregulates the secretion of these enzymes to initiate its own defense mechanisms against *F. solani* infection.

It has been reported that taurine may promote the synthesis and enhance the activity of defense enzymes by modulating signaling pathways and activating the expression of defense-related genes within plant cells [52]. In addition to its direct inhibitory effect on pathogens, methyl cinnamate may also act as a signaling molecule to induce systemic resistance in *A. sinensis*, thereby increasing the activity of defense enzymes [53]. The defense enzyme activities in the B-2 group were essentially comparable to those in the DP group, indicating that this substance does not significantly activate the active defense of *A. sinensis*. It likely maintains the growth and basic resistance of *A. sinensis* primarily through other pathways, particularly given that 2-phenylethanol has been confirmed to possess significant antimicrobial effects. The defense enzyme activities in the R-3HB group and the OXY group were slightly higher than those in the CK group, while the enzyme activities in the Q group and the P-6 group were generally similar to the CK group but markedly lower than those in the T and EMC groups. This suggests that although these metabolic intermediates can trigger defense responses in *A. sinensis* to some extent, their effects are relatively weak (with phenylalanine ammonia-lyase activity being specifically enhanced due to the presence of 6-phosphogluconic acid).

The changes in defense enzyme activities are closely linked to the disease resistance of *A. sinensis*. Studies have shown that POD is involved in redox reactions, catalyzing the decomposition of hydrogen peroxide to scavenge intracellular ROS. It also participates in lignin synthesis, strengthening the cell wall to block pathogen invasion [54]. PAL is a key enzyme in the phenylpropanoid pathway, catalyzing the conversion of phenylalanine to trans-cinnamic acid, which leads to the synthesis of a series of defense-related secondary metabolites such as lignin and phytoalexins, thereby enhancing plant disease resistance [55]. LOX plays crucial roles in plant growth, development, fruit ripening, and response to environmental stresses as a key enzyme in lipid metabolism [56]. PPO, a copper-containing oxidoreductase, is significant in plant processes including photosynthesis, catalysis of phenolic compounds, and defense against environmental stress [57]. Therefore, the higher defense enzyme activities observed in the T and EMC groups likely constitute one of the key reasons for their efficacy in suppressing *F. solani*-induced disease and maintaining normal growth in *A. sinensis*.

#### 4.3.3. Regulation of Rhizosphere Microorganisms

Different “cry for help” metabolites significant effects on the composition and structure of the microbial community in *A. sinensis* rhizosphere soil. Regarding the bacterial community, the dominant bacterial composition at the phylum level was largely consistent across the nine treatment groups. Ten phyla, including Proteobacteria, *Bacteroidota*, and *Actinobacteria*, collectively accounted for an average relative abundance exceeding 96.71% of the total, although certain inter-group differences in relative abundance were observed. Specifically, the relative abundance of *Actinobacteria* in the T group (taurine) was significantly higher compared to the CK and DP groups. *Actinobacteria* are a group of microorganisms with important ecological functions. Many actinobacteria can produce bioactive substances such as antibiotics and enzymes, which exhibit inhibitory effects against pathogens, exemplified by genera like *Streptomyces* [58], *Micromonospora* [59], *Arthrobacter* [60], and *Actinomyces* [61]. Simultaneously, *Actinobacteria* also engage in the decomposition of organic matter and nutrient cycling in soil, thereby improving the soil ecological environment [62]. Therefore, the increased relative abundance of *Actinobacteria* in the T group may contribute to enhancing the antibacterial capacity of the *A. sinensis* rhizosphere soil and promoting the healthy growth of the plants. In the EMC group (methyl cinnamate), the relative abundances of *Bacteroidota* and *Proteobacteria* were significantly elevated. *Bacteroidota* play a crucial role in the decomposition and transformation of soil organic matter, capable of breaking down complex organic compounds into simpler ones, thereby providing plants with accessible nutrients [63]. *Proteobacteria* encompass numerous bacteria closely associated with plant growth and health, such as nitrogen-fixing bacteria [64] and rhizobia [65], which participate in the nitrogen cycle, supply nitrogen to plants, and promote plant growth [66].

At the genus level, distinct differences were observed in the dominant bacterial genera of the rhizosphere soil of *A. sinensis* among the nine treatment groups. In the T group, the relative abundance of *Paeniglutamicibacter* reached as high as 22.1%, significantly exceeding that in other treatments. Current studies indicate that some strains of Paeniglutamicibacter can promote plant growth and enhance resistance to heavy metal stress [67]; however, research on this genus remains relatively limited, and whether *Paeniglutamicibacter* strains possess the ability to resist pathogen infection awaits further investigation. In the EMC group, the relative abundance of *Azotobacter* was as high as 21.4%, and this genus was not detected among the top 10 dominant genera in the other eight treatment groups. Azotobacter can convert atmospheric nitrogen into plant-available ammonia, thereby providing sufficient nitrogen nutrition for *A. sinensis* [68]. Furthermore, *Pseudomonas*, *Devosia*, and an unclassified genus of *Rhizobiaceae* were among the top 10 dominant genera in most treatment groups, representing the core shared dominant bacterial genera in the *A. sinensis* rhizosphere. These genera may play important synergistic roles in plant growth promotion and disease suppression.

Regarding the fungal community, the composition of dominant fungi at the phylum level was largely conserved across the nine treatment groups. The relative abundances of ten phyla, including *Ascomycota* and *Basidiomycota*, collectively accounted for an average of over 99.79% of the total sequences, albeit with inter-group variations in their relative proportions. In the T group, the relative abundance of *Ascomycota* was markedly reduced, whereas that of *Chytridiomycota* increased significantly. It is reported that a majority of phytopathogenic fungi belong to *Ascomycota* [69], such as *Magnaporthe oryzae*, *Botrytis cinerea*, *Fusarium graminearum*, *Fusarium oxysporum*, *Blumeria graminis*, *Mycosphaerella graminicola*, and *Colletotrichum* spp. [70]. The decrease in *Ascomycota* relative abundance in the T group suggests a potential reduction in pathogenic load. For the increase in *Chytridiomycota*, however, the underlying mechanisms remain unclear but may be related to its role in regulating the soil ecological environment or through interactions with other microorganisms. However, it should be noted that the taurine (T) group enriched multiple opportunistic pathogenic taxa, including the known plant pathogen *Plectosphaerella* (Table 7). Although taurine reduced the relative abundance of *Ascomycota* (to which many phytopathogenic fungi belong) and inhibited *F. solani*, it simultaneously posed a potential ecological risk by enriching other opportunistic pathogens in the rhizosphere. This trade-off suggests that *F. solani* may not only directly infect *A. sinensis* roots but also alter the rhizosphere microenvironment to recruit and enrich other pathogenic microorganisms, potentially forming a synergistic pathogenic consortium that exacerbates root rot damage. In contrast, methyl cinnamate profoundly restructured the rhizosphere microbial community: it inhibited the proliferation of harmful pathogens induced by *F. solani* and avoided the enrichment of opportunistic pathogens observed in the taurine group, while selectively recruiting beneficial plant growth-promoting and biocontrol microbes. Future studies should assess the long-term ecological consequences of taurine application and determine whether co-application with methyl cinnamate could mitigate this unintended effect. In the EMC group, the relative abundance of *Ascomycota* was significantly elevated, within which the genus *Schizothecium* showed a notable increase compared to the diseased group. *Schizothecium* is a saprophytic fungal genus that may play a role in modulating the rhizosphere microbial community structure and suppressing the growth of detrimental fungi [71]. The relative abundance of *F. solani* decreased across all treatment groups compared to the diseased group, with the most pronounced declines observed in treatments T and EMC. This further indicates that taurine and methyl cinnamate can directly or indirectly inhibit the growth and proliferation of *F. solani* in the rhizosphere soil of *A. sinensis*.

Changes in the rhizosphere microbial community are closely associated with the health status of *A. sinensis*. The enrichment of beneficial microbes can enhance soil suppressiveness, promote plant nutrient acquisition, and improve plant stress resistance. Conversely, the depletion of harmful microorganisms directly reduces the risk of disease occurrence. Therefore, different “cry for help” compounds play crucial roles in maintaining the healthy growth of *A. sinensis* by modulating the composition and structure of its rhizosphere microbial community.

## 5. Conclusions

This study systematically revealed the metabolic characteristics of *F. solani*, clarified its impact on the metabolome of the *A. sinensis* root zone, and investigated the regulatory effects of potential “cry for help” compounds on the growth phenotype, defensive enzymes, and rhizosphere microbiota of *A. sinensis*. Notably, the taurine group enriched opportunistic pathogenic taxa, highlighting a potential ecological risk warranting further investigation, and suggesting that *F. solani* may recruit other pathogens to form a synergistic consortium that exacerbates root rot damage. Combined with the metabolic profiling of *F. solani* and the physiological function of methyl cinnamate, this compound was inferred to serve as a core “cry for help” signal molecule of *A. sinensis* in response to *F. solani* stress.

The results preliminarily identified taurine and methyl cinnamate as key “cry for help” compounds in *A. sinensis* under *F. solani* stress. Both compounds significantly enhanced the activities of defense-related enzymes compared to the diseased control, with EMC showing consistently higher levels of PAL, LOX, and PPO than T. EMC profoundly restructured the rhizosphere microbial community: it inhibited the proliferation of harmful pathogens induced by *F. solani*, avoided the enrichment of opportunistic pathogens observed in the T group, and selectively recruited beneficial plant growth-promoting and biocontrol microbes. In contrast, although T suppressed *F. solani*, it simultaneously enriched opportunistic pathogenic taxa such as *Plectosphaerella*, representing a potential ecological trade-off that warrants further investigation. It is worth emphasizing that although taurine can activate the defense response of A. sinensis and inhibit the growth of F. solani to a certain extent, its significant enrichment of opportunistic pathogenic taxa implies potential phytotoxicity and ecological side effects. Therefore, subsequent biological verification experiments on taurine need to be more rigorous and comprehensive. It is necessary to systematically evaluate its dual effects on A. sinensis and rhizosphere microorganisms, strictly verify whether it will induce secondary biological stress on A. sinensis while exerting antibacterial effects, and avoid aggravating root rot and other adverse diseases of A. sinensis, so as to ensure the safety and effectiveness of taurine as a potential regulatory compound. Collectively, EMC exhibited more efficient and comprehensive protective effects by enhancing defense enzyme activity and reconstructing the rhizosphere microbiota. These findings provide a novel perspective for understanding plant–pathogen interaction mechanisms and establish a theoretical foundation for biocontrol strategies based on “cry for help” compounds. Our research group will conduct further validation experiments to elucidate the functions of key microorganisms recruited by these metabolites, aiming to provide new insights for the prevention and control of *A. sinensis* root rot.

## Figures and Tables

**Table 1:** Effect of the “cry for help” metabolite mixture on the growth indices of *A. sinensis*.

Treatments	Root Length (cm)	Underground Biomass (g)	Main Root Diameter (mm)	Aboveground Biomass (g)
1:1	11.80 ± 0.55 a	0.928 ± 0.027 a	6.41 ± 0.36 a	0.321 ± 0.035 a
1:2	9.43 ± 0.57 b	0.591 ± 0.058 b	5.03 ± 0.23 b	0.181 ± 0.006 b
1:3	8.97 ± 1.02 b	0.487 ± 0.244 c	4.86 ± 0.16 c	0.199 ± 0.015 c
DP	8.17 ± 0.26 c	0.495 ± 0.105 c	5.15 ± 0.31 b	0.200 ± 0.040 b

Note: Values are presented as mean ± SD. Different lowercase letters within the same column indicate significant differences (*p* < 0.05).

**Table 2:** Effects of different treatments on the growth indices of *A. sinensis*.

Treatments	Plant Height (cm)	Root Length (cm)	Underground Biomass (g)	Main Root Diameter (mm)	Aboveground Biomass (g)	Disease Index
CK	16.20 ± 0.43 ab	8.97 ± 0.62 a	0.698 ± 0.094 ab	5.52 ± 0.16 ab	0.282 ± 0.032 ab	0.00 ± 0.00 d
DP	13.59 ± 0.32 c	6.46 ± 0.17 d	0.416 ± 0.101 d	4.65 ± 0.23 d	0.179 ± 0.022 d	66.67 ± 2.54 a
Q	14.27 ± 0.36 c	7.18 ± 0.41 cd	0.468 ± 0.074 cd	4.95 ± 0.11 cd	0.183 ± 0.029 d	46.67 ± 1.26 b
T	18.36 ± 0.77 a	9.24 ± 0.42 a	0.917 ± 0.116 a	6.18 ± 0.31 a	0.311 ± 0.041 a	15.33 ± 0.82 c
EMC	17.72 ± 0.65 a	9.46 ± 0.37 a	0.952 ± 0.101 a	6.05 ± 0.33 a	0.277 ± 0.052 ab	15.83 ± 0.75 c
R-3 HB	15.26 ± 0.39 b	7.90 ± 0.22 b	0.673 ± 0.092 bc	5.13 ± 0.21 bc	0.246 ± 0.017 bc	43.33 ± 0.90 b
B-2	16.46 ± 0.38 ab	8.92 ± 0.46 a	0.696 ± 0.088 ab	5.62 ± 0.24 ab	0.244 ± 0.023 bc	36.67 ± 0.85 bc
OXY	14.80 ± 0.28 bc	8.17 ± 0.32 bc	0.648 ± 0.104 bc	5.13 ± 0.34 bc	0.254 ± 0.033 bc	55.00 ± 0.54 ab
P-6	16.33 ± 0.33 a	8.84 ± 0.36 a	0.663 ± 0.080 bc	5.22 ± 0.62 bc	0.241 ± 0.019 bc	30.00 ± 0.42 bc

Note: Values are presented as mean ± SD. Different lowercase letters within the same column indicate significant differences (*p* < 0.05).

**Table 3:** Effect of Different Treatments on the Defense-Related Enzyme Activities in *A. sinensis*.

Treatments	POD (U/g)	PAL (U/g)	LOX (U/g)	PPO (U/g)
CK	1.84 ± 0.11 b	0.11 ± 0.003 b	1.84 ± 0.08 b	14.61 ± 0.11 d
DP	2.15 ± 0.05 a	0.13 ± 0.005 a	2.31 ± 0.14 a	16.78 ± 0.34 b
Q	1.91 ± 0.08 b	0.13 ± 0.004 b	2.00 ± 0.12 b	14.93 ± 0.20 d
T	2.20 ± 0.09 a	0.15 ± 0.007 a	2.67 ± 0.18 a	17.75 ± 0.61 a
EMC	2.44 ± 0.12 a	0.19 ± 0.008 a	3.03 ± 0.20 a	18.97 ± 0.88 a
R-3HB	2.07 ± 0.06 a	0.14 ± 0.005 b	2.07 ± 0.12 b	15.45 ± 0.39 c
B-2	2.14 ± 0.10 a	0.15 ± 0.006 a	2.42 ± 0.13 a	15.81 ± 0.34 c
OXY	2.07 ± 0.06 a	0.13 ± 0.002 b	2.03 ± 0.11 b	15.00 ± 0.20 d
P-6	2.01 ± 0.04 a	0.21 ± 0.002 b	1.93 ± 0.12 b	14.77 ± 0.14 d

U/g represents the amount of enzyme required to catalyze the conversion of 1 μmol substrate per minute under assay conditions. All enzyme activities are expressed in units of U/g fresh weight. Note: Values are presented as mean ± SD. Different lowercase letters within the same column indicate significant differences (*p* < 0.05).

**Table 4:** Changes in Soil Bacterial Alpha Diversity Indices of *Angelica sinensis* Rhizosphere under *Fusarium solani* Stress.

Samples	Ace Index	Chao1 Index	Shannon Index	Simpson Index
CK	1202.67 ± 57.61 a	1203.43 ± 59.59 a	6.44 ± 0.06 a	0.0029 ± 0.0005 a
DP	1270.32 ± 151.03 b	1270.23 ± 151.46 b	6.42 ± 0.14 a	0.0040 ± 0.0010 b
EMC	1090.85 ± 57.99 bc	1090.64 ± 58.05 bc	5.87 ± 0.21 b	0.0180 ± 0.0085 bc
T	916.04 ± 120.59 cd	915.80 ± 120.70 cd	5.74 ± 0.40 bc	0.0144 ± 0.0058 c
B-2	1093.78 ± 9.09 de	1094.83 ± 9.28 de	5.78 ± 0.51 b	0.0211 ± 0.0149 bc
Q	1124.61 ± 58.94 cd	1124.45 ± 59.33 cd	6.25 ± 0.15 c	0.0053 ± 0.0018 d
R-3 HB	976.99 ± 65.25 cd	976.66 ± 65.61 cd	5.46 ± 0.41 cd	0.0367 ± 0.0207 e
P-6	1289.48 ± 143.12 bc	1289.06 ± 142.99 bc	6.44 ± 0.10 bc	0.0034 ± 0.0003 de
OXY	1251.79 ± 102.60 b	1251.55 ± 102.61 b	6.44 ± 0.12 a	0.0033 ± 0.0007 de

Note: Values are presented as mean ± SD. Different lowercase letters within the same column indicate significant differences (*p* < 0.05).

**Table 5:** Composition of Dominant Bacterial Genera in the Rhizosphere Soil of *A. sinensis* Under Different Treatments.

Treatments	Dominant Bacterial Genera (Relative Abundance/%)					
CK	Rhizobacter (7.2%)	Sphingomonas (6.5%)	Pseudomonas (3.1%)	Methylocaldum (2.4%)	Rummeliibacillus (2.3%)	Bacillus (2.0%)
DP	Sphingomonas (5.4%)	Pseudomonas (4.2%)	Paeniglutamicibacter (3.5%)	Methylocaldum (2.9%)	Rhizobiaceae_unclassified (2.6%)	Micrococcaceae_unclassified (2.0%)
Q	Micrococcaceae_unclassified (8.4%)	Planococcus (4.7%)	JG30-KF-CM45 (2.8%)	Luteimonas (2.3%)	Devosia (1.9%)	Cellvibrio (1.8%)
T	Paeniglutamicibacter (22.1%)	Micrococcaceae_unclassified (5.9%)	Rummeliibacillus (1.9%)	Lysobacter (1.5%)	JG30-KF-CM45 (1.5%)	Rhizobiaceae_unclassified (1.4%)
EMC	Azotobacter (21.4%)	Bacillus (2.7%)	Pseudomonas (2.4%)	Rhizobiaceae_unclassified (2.3%)	Devosia (1.9%)	Methylocaldum (1.6%)
R-3HB	Cellvibrio (4.8%)	Devosia (4.6%)	Bacillus (3.2%)	Rhizobiaceae_unclassified (3.2%)	Pseudomonas (2.2%)	Planococcus (1.7%)
B-2	Planococcus (4.3%)	Rhizobiaceae_unclassified (2.9%)	Pseudomonas (2.5%)	Devosia (2.4%)	Bacillus (2.2%)	JG30-KF-CM45 (1.8%)
OXY	Cellvibrio (4.4%)	Bacillus (3.3%)	Rhizobiaceae_unclassified (3.2%)	Devosia (2.6%)	Luteimonas (2.3%)	Paeniglutamicibacter (2.1%)
P-6	Planifilum (5.0%)	Methylocaldum (2.7%)	Rhizobiaceae_unclassified (2.1%)	Cellvibrio (2.0%)	Rummeliibacillus (1.9%)	Devosia (1.5%)

**Table 6:** Changes in Soil Fungal Alpha Diversity Indices of *Angelica sinensis* Rhizosphere under *Fusarium solani* Stress.

Samples	Ace Index	Chao1 Index	Shannon Index	Simpson Index
CK	202.20 ± 13.82 a	202.09 ± 13.83 a	3.28 ± 0.14 a	0.084 ± 0.013 a
DP	213.08 ± 39.88 ab	212.81 ± 39.97 ab	3.26 ± 0.66 ab	0.105 ± 0.102 b
EMC	174.46 ± 36.73 bc	174.33 ± 36.64 c	3.16 ± 0.46 b	0.119 ± 0.084 bc
T	165.62 ± 33.84 c	165.43 ± 33.77 cd	3.10 ± 0.27 ab	0.092 ± 0.018 ab
B-2	167.36 ± 29.91 c	167.05 ± 29.77 cd	3.02 ± 0.70 c	0.128 ± 0.105 cd
Q	229.40 ± 30.44 d	229.22 ± 30.52 b	3.45 ± 0.22 d	0.064 ± 0.014 e
R-3HB	208.49 ± 12.93 cd	208.27 ± 12.82 ab	3.48 ± 0.03 d	0.070 ± 0.015 de
P-6	181.50 ± 27.78 bc	181.29 ± 27.78 c	3.24 ± 0.36 ab	0.087 ± 0.044 cde
OXY	223.64 ± 22.25 b	223.48 ± 22.28 ab	3.60 ± 0.17 d	0.058 ± 0.015 e

Note: Values are presented as mean ± SD. Different lowercase letters within the same column indicate significant differences (*p* < 0.05).

**Table 7:** Composition of dominant fungal genera in the Rhizosphere Soil of *A. sinensis* Under Different Treatments.

Treatments	Dominant Fungal Genera (Relative Abundance/%)					
CK	Coprinus (25.1%)	Schizothecium (17.1%)	Thermomyces (14.4%)	Aspergillus (5.2%)	Iodophanus (2.1%)	Stachybotrys (2.0%)
DP	Coprinus (27.9%)	Plectosphaerella (14.7%)	Fusarium (10.1%)	Sordariaceae_unclassified (4.9%)	Olpidiaster (6.8%)	Gaertneriomyces (6.5%)
Q	Coprinus (17.5%)	Thermomyces (15.4%)	Schizothecium (8.3%)	Sordariaceae_unclassified (7.4%)	Plectosphaerella (7.0%)	Fusarium (6.8%)
T	Coprinus (21.1%)	Plectosphaerella (8.6%)	Olpidiaster (7.1%)	Sordariaceae_unclassified (5.9%)	Olpidiaster (4.3%)	unclassified (4.0%)
EMC	Schizothecium (38.8%)	Plectosphaerella (9.9%)	Peziza (9.6%)	Coprinus (5.7%)	Fungi_gen_Incertae_sedis (4.0%)	Thermomyces (3.0%)
R-3HB	Coprinus (17.5%)	Thermomyces (15.7%)	Schizothecium (8.3%)	Sordariaceae_unclassified (7.3%)	Plectosphaerella (7.0%)	Fusarium (6.9%)
P-6	Coprinus (21.7%)	Thermomyces (15.0%)	Schizothecium (14.3%)	Plectosphaerella (8.6%)	Fusarium (6.2%)	Gaertneriomyces (4.5%)
B-2	Coprinus (28.9%)	Iodophanus (15.3%)	Schizothecium (9.7%)	Thermomyces (6.7%)	Fusarium (5.6%)	Stachybotrys (4.5%)
OXY	Coprinus (27.4%)	Schizothecium (14.2%)	Sordariaceae_unclassified (12.0%)	Thermomyces (7.0%)	Stachybotrys (5.5%)	Fusarium (5.1%)

## Data Availability

Due to the excessively large size of the raw data generated in this study, it cannot be uploaded to MetaboLights and SRA. Please contact the corresponding author for access to the raw data if you require it for verification purposes.

## Abbreviations

The following abbreviations are used in this manuscript:

PCR	Polymerase chain reaction
PLS-DA	Partial Least Squares Discriminant Analysis
ASVs	Amplicon sequence variants
PDA	Potato dextrose agar
PCA	Principal component analysis
